# The Anti-social Brain in Schizophrenia: A Role of CaMKII?

**DOI:** 10.3389/fpsyt.2022.868244

**Published:** 2022-05-30

**Authors:** Rana El Rawas, Inês M. Amaral, Alex Hofer

**Affiliations:** Division of Psychiatry I, Department of Psychiatry, Psychotherapy, Psychosomatics and Medical Psychology, Medical University Innsbruck, Innsbruck, Austria

**Keywords:** schizophrenia, CaMKII, social interaction, negative symptoms, biomarker

## Abstract

Current pharmacological therapy has limited effects on the cognitive impairments and negative symptoms associated with schizophrenia. Therefore, understanding the molecular underpinnings of this disorder is essential for the development of effective treatments. It appears that a reduction in calcium/calmodulin-dependent protein kinase II (α-CaMKII) activity is a common mechanism underlying the abnormal social behavior and cognitive deficits associated with schizophrenia. In addition, in a previous study social interaction with a partner of the same sex and weight increased the activity of α-CaMKII in rats. Here, we propose that boosting of CaMKII signaling, in a manner that counteracts this neuropsychiatric disease without disrupting the normal brain function, might ameliorate the abnormalities in social cognition and the negative symptoms of schizophrenia.

## Introduction

Schizophrenia is a complex, chronic, and polygenic neuropsychiatric disorder that affects more than one percent of the world adult population. Typical clinical manifestations are positive symptoms (e.g., hallucinations, delusions, disordered thoughts, and speech), negative symptoms (e.g., deficits in social interaction, diminished expression and motivation, anhedonia, apathy), and deficits in both neurocognition (processing speed, attention/vigilance, working memory, verbal learning and memory, visual learning and memory, reasoning and problem solving, verbal comprehension, and verbal fluency) and social cognition (emotional processing, social perception and knowledge, theory of mind, and attributional bias). Notably, negative and cognitive symptoms have a larger impact on patients’ functioning than positive symptoms (1) and correlate with the degree of disability (2, 3).

Around 20–35% of the people affected by schizophrenia fail to respond to antipsychotics (4) and current pharmacological therapy has limited effects on cognitive impairments and negative symptoms (5). Moreover, existing treatments reduce the severity of symptoms rather than providing a cure. Therefore, understanding the molecular mechanisms underlying schizophrenia is essential for the development of effective treatments.

For many years, the essential role of dopamine in the pathogenesis of schizophrenia has been proposed for the reasons that all currently available antipsychotic agents target hyperdopaminergia in the brain via postsynaptic dopamine receptor blockade (6) and that in humans, dopamine-like agents such as amphetamine mimic the positive symptoms of schizophrenia (7). On the other hand, phencyclidine (PCP) and ketamine, both non-competitive N-methyl-D-aspartate (NMDA) receptor antagonists, induce schizophrenia-like psychosis (8), thereby supporting a “glutamatergic” implication in the pathophysiology of schizophrenia. Since then, many researchers have suggested that insufficient glutamate neurotransmission is involved in this disorder (9). Particularly, it has been proposed that dysfunction in calcium/calmodulin-dependent protein kinase II (CaMKII) expression and activity is a common mechanism underlying changes in glutamatergic structural and functional synaptic plasticity that may directly contribute to neuropsychiatric diseases (10).

Calcium/calmodulin-dependent protein kinase II is a serine/threonine kinase found throughout the brain (11, 12) and is activated upon Ca^2+^/calmodulin (CaM) binding. This kinase has a key role in synaptic signaling and consequently in learning and memory, not only due to its cellular and subcellular location, but also due to the time-course of its activity and autophosphorylation properties (10, 12, 13). In mammals, CaMKII subunits are encoded by closely related gene products—α, β, γ, and δ. Of note, CaMKII α and β isoforms are predominant in the brain (11).

Each CaMKII isoform comprises (1) an N-terminal catalytic domain that contains the ATP- and the substrate-binding (S) sites, (2) an auto-inhibitory regulatory domain that includes a pseudo-substrate segment and a threonine residue 286 (Thr286) segment (or Thr287, depending on the CaMKII isoform), and (3) a C-terminal association domain. The auto-inhibitory and catalytic domains form a gate that regulates activity in a way that when these domains bind to each other at the S site (binding to the pseudo-substrate region) and at a site known as T (binding to the region around Thr286) of the catalytic domain, the enzyme is inhibited and the gate is closed (12). The association domain is required for oligomerization (13, 14). This region is linked to the catalytic and regulatory domains by a variable region that is responsible for most of the structural differences between isoforms (12).

In the presence of Ca^2+^, the Ca^2+^/CaM complex can bind to CaMKII on a region that overlaps with the pseudo-substrate region, opening the gate and inducing a conformational change that will expose the catalytic domain and thus activate CaMKII (14). A site on the NMDA receptor NR2B subunit can bind to the T side, keeping the gate open and the enzyme active even after the dissociation of calmodulin (12). In the presence of Ca^2+^/CaM, the Thr286 residue on the auto-inhibitory domain of α-CaMKII (or Thr287 on βCaMKII) can become autophosphorylated by a neighboring, activated subunit (14). Even when intracellular Ca^2+^ levels decrease and CaM dissociates from its complex, the inter-subunit autophosphorylation prevents CaMKII from reverting back to its inactive state (12), acquiring autonomous and Ca^2+^-independent activity. De-phosphorylation returns the enzyme to an inactive state and is catalyzed by protein phosphatase types 1 and 2A (14).

## CaMKII: A Biomarker for Schizophrenia?

**FIGURE 1:**
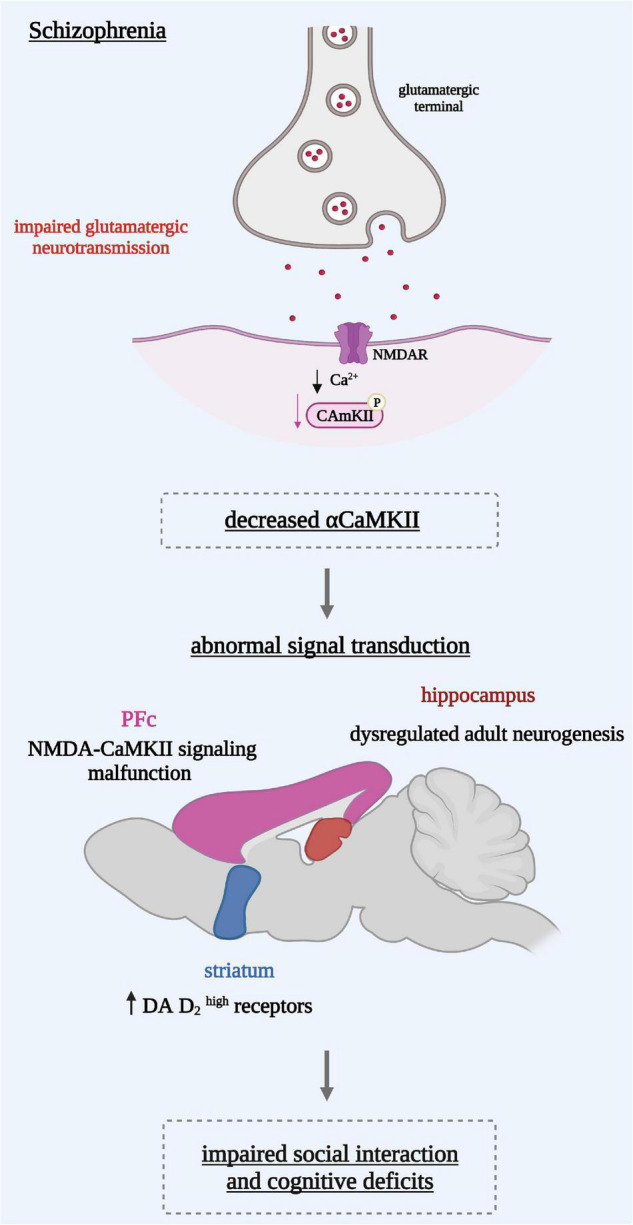
Reduced α-CaMKII induces on the cellular level an abnormal signal transduction reflected in different brain regions. In the hippocampus: a dysregulated adult neurogenesis leading to an immature dentate gyrus in the hippocampus. In the pre-frontal cortex (PFc): a malfunction of NMDA receptor signaling in the associated with dopaminergic hypo-function. In the striatum: dopamine (DA) D2 receptors in a state with a high affinity for DA leading to a hyperdopaminergic state. These mechanisms might underlie behavioral abnormalities such as the social interaction impairments and cognitive deficits seen in schizophrenia.

To model the pathophysiology of schizophrenia, many transgenic mouse lines have been generated. In addition, other animal models based on pharmacological manipulations of the glutamatergic or the dopaminergic system have been explored. It is perceived that reduced CaMKII function (Figure 1) could be a common mechanism for various symptoms observed in schizophrenia (10).

The most prominent behavioral phenotypes were those carrying a heterozygous null mutation for α-calcium/calmodulin kinase II or α-CaMKII ^+/−^ mice. These mice showed features analogous to the ones found in schizophrenia. Most notably, they showed increased locomotor activity, a severe working memory deficit, disrupted circadian activity, and social withdrawal in addition to high levels of aggression toward cage mates (15–17). Moreover, transcriptome analysis and comprehensive autoradiography studies indicated that the mice had marked abnormalities in gene expression and receptor binding in the hippocampus, specifically in the dentate gyrus (DG) (16) where adult neurogenesis partially occurs (18). Whereas the number of newborn neurons in the mutant DG mice was increased by more than 50%, the number of mature neurons was intensely decreased in a way that the DG neurons in the α-CaMKII ^+/−^ mice were mostly containing immature neurons leading to an “immature DG” (16).

It has been suggested that adult neurogenesis has a potential role in psychiatric disorders including schizophrenia (19). Indeed, dysregulated adult neurogenesis has been associated with neurocognitive impairments in forms of learning and memory (20), and abnormal hippocampal function (17). In line with these facts, α-CaMKII ^+/−^ mice exhibited specific learning impairments, in particular in regards of spatial learning (21).

On the other hand, the levels of dopamine (DA) D2 receptors in a state with a high affinity for DA (D2 ^high^ receptors) were found to be elevated in the striatum of α-CaMKII ^+/−^ mice, thereby reflecting the hyperdopaminergic state seen in patients with schizophrenia. This high affinity state of DA D2 receptors might possibly be a consequence of elevated β-CaMKII mRNA levels observed in the striatum of these mice with reduced α-CaMKII expression (22), which is probably a compensatory effect. Elevated β-CaMKII subunit mRNA expression in rats’ striatum was also found in the amphetamine sensitization animal model of psychosis (23). In addition, these hyperactive animals show elevated levels of D2 ^high^ receptors (22). Remarkably, the CaMKII inhibitor, KN-93, markedly reduced the D2 ^high^ states in the rat striatum (22), suggesting that β-CaMKII may increase the D2 ^high^ receptors state in the striatum of animals and possibly in schizophrenia (22).

Phencyclidine, a non-competitive NMDA antagonist, reproduces a schizophrenia-like psychosis including positive and negative symptoms as well as cognitive deficits. PCP treated mice have been shown to exhibit hyperlocomotion as an index of positive symptoms, negative symptoms reflected by an enhanced immobility in a forced swimming test, and reduced social interaction and cognitive deficits revealed by impairments of latent learning in a water finding test and recognition memory (24–26). In these mice, α-CaMKII phosphorylation (Thr286) was reduced in the prefrontal cortex (PFc) in comparison to control mice (24, 25, 27, 28). As behavioral impairments and abnormal intracellular signaling were alleviated after potentiation of NMDA receptor function, it has been suggested that repeated PCP treatment induces dysfunction of NMDA-CaMKII signaling in the PFc (24, 28). Moreover, the animals treated repeatedly with PCP failed to release DA in response to high potassium stimulation or a challenge of PCP in the PFc (28). Thus, it is possible that repeated PCP treatment induces a malfunction of NMDA-CaMKII signaling in the PFc, which is associated with dopaminergic hypo-function (28).

This uniquely well situated substrate, predominantly located in the postsynaptic density of excitatory glutamatergic neurons (29), appears to be a common actor in schizophrenia. In other animal models of this disorder, in particular ketamine-treated mice were shown to exhibit deficiencies in sociability and social novelty behavior associated with a significant decrease in hippocampal α-CaMKII expression (30). Previous studies indicate that post-pubertal neonatal ventral hippocampal lesioned rats exhibit impairments in prepulse inhibition (PPI), spontaneous locomotion, social interaction behavior, and working memory (31, 32). In these animals, CaMKII autophosphorylation is significantly reduced, especially in the medial PFc, the striatum, and the hippocampal CA1 region relative to control animals (31, 32). In a model of early life stress, α-CaMKII was found to be downregulated in the PFc (33). Furthermore, late adolescent stress in combination with disrupted-in-Schizophrenia 1 (DISC1) genetic risk impaired activation of NMDA-Ca^2+^/calmodulin kinase II signaling in the PFc (34), resulting in impaired social interaction and novelty preference for object recognition memory (34). In dysbindin-1-deficient mice, reduced levels of CaMKII were reported in the medial PFc (35). Notably, dysbindin-1 in the PFc has also been shown to be reduced in schizophrenia patients (36, 37). This reduction is thought to promote NMDA receptor hypo-function, thereby leading to the cognitive deficits observed in schizophrenia (37).

Altogether, these findings indicate that the α-CaMKII ^+/−^ mouse and others like it may provide genetic biomarkers that can be used to improve treatments for schizophrenia (10). In Table 1, we summarize the findings reporting that dysregulated CaMKII signaling causes impaired social interaction and cognitive deficits.

**TABLE 1:** Summary of the findings reporting that dysregulated CaMKII signaling causes impaired social interaction and cognitive deficits.

Treatment/model	Molecular	Behavior	References
α-CaMKII ^+/−^ mice	↓hippocampus α-CaMKII, ↓frontal cortex α-CaMKII mRNA, ↑striatum β-CaMKII mRNA, ↑striatum D2 ^high^ receptors	Social withdrawal, Severe working memory deficits, Profound impairment in learning tasks, Hyperactivity, Exagerated infradian rythm	(15, 16, 21, 22)
PCP-treated mice	↓ PFc p (Thr 286) α-CaMKII	Social deficits, Memory impairments, Impairment of latent learning, Increased imobility in forced swim test	(24, 25)
Ketamine-treated mice	↓hippocampus α-CaMKII	Decrease in sociability and social novelty behavior	(30)
Neonatal lesion of ventral hippocampus	↓mPFc, striatum, and Hippocampus CA1 region CaMKII autophosphorylation	Impairments in prepulse inhibition (PPI), Spontaneous locomotion, Social interaction behavior and working memory	(31, 32)
Late adolescent stress in combination with DISC1 genetic risk	Impaired activation of NMDA-Ca^2+^/calmodulin kinase II signaling in the PFc	Deficits in locomotor activity, Forced swim, Social interaction, and Novelty preference tests	(34)
Autophosphorylation deficient (α-CaMKII-Thr286A) mice		Decreased social preference and interest in conspecifics of the same sex, Decreased levels of social interactions in a social group	(38)
*de novo* Glu183 to Val (E183V) mutation in the CaMKIIα catalytic domain (CaMKIIα-E183V) mice	↓ forebrain α-CaMKII	Hyperactivity, Social interaction deficits, and Increased repetitive behaviors	(39)

*↑ increase; ↓ decrease; PFc, prefrontal cortex.*

## Which Behavior to Focus On?

As is the case with many psychiatric disorders and as mentioned above, schizophrenia is characterized by different symptoms: positive symptoms, negative symptoms, and cognitive impairments. Evidently, some of these symptoms are uniquely human and impossible to model in an animal (17). Importantly, the vast majority of the animal models commonly share the profile of impaired social interaction. This behavioral abnormality seems to be tightly linked to CaMKII. Indeed, autophosphorylation-deficient (α-CaMKII-Thr286A) mutant female mice show abnormal social behaviors characterized by decreased social preference and interest in conspecifics of the same sex, as compared to controls (38). Moreover, these mutant mice show decreased levels of social interactions in a social group, as compared to control mice (38). Whereas, control mice increase the frequency of close social interactions during a learning task, α-CaMKII-T286A mutant mice do not (38). In line with these findings, mice with a mutation in the CaMKII-α catalytic domain having lower total forebrain CaMKII-α levels, display aberrant behavioral phenotypes, in particular social interaction deficits (39).

**FIGURE 2:**
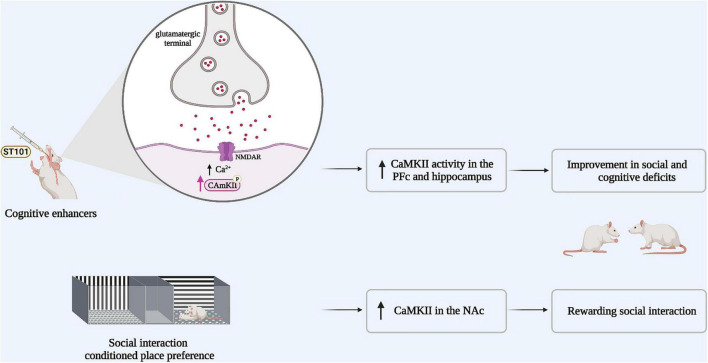
Boosting CaMKII signaling could improve both social and cognitive deficits in schizophrenia. Animal models of schizophrenia share the same behavioral profile, in particular social withdrawal. Impaired social interaction is associated with reduced α-CaMKII activity. If CaMKII activity was potentiated via the administration of cognitive enhancers such as (ST101), CaMKII activity in the pre-frontal cortex (PFc) and the hippocampus is increased and the social impairment is rescued. In parallel, social interaction reward increases α-CaMKII activity in the nucleus accumbens (NAc).

In a recent study, a conditioned place preference (CPP) to a social interaction partner of the same sex, age, and weight was shown to increase α-CaMKII activity in the nucleus accumbens (NAc) (40). In the CPP paradigm, the animal learns to associate a stimulus with a specific context during conditioning, and if this stimulus is appetitive, the animal will prefer to spend more time in the context associated with this stimulus when the choice to “prefer” between a stimulus or a neutral-associated context is given. This study also demonstrated that inhibition of CaMKII in the NAc shell decreases the preference for social interaction (40). These results suggest that social interaction reward is associated with an increased α-CaMKII phosphorylation in this region (Figure 2).

Rats with a neonatal ventral hippocampus lesion exhibit impaired social interaction and reduced CaMKII signaling in memory-related brain regions, resistant to second generation antipsychotics such as risperidone (32, 41). Notably, the administration of the cognitive enhancer spiro[imi-dazo[1,2-a]pyridine-3,2-indan]-2(3H)-one (ST101), an enhancer of T-type calcium channels (42, 43), stimulates CaMKII activity in the hippocampus and the medial PFc and significantly improves deficits in social interaction and cognitive function in these rats (43). It has therefore been proposed that ST101 may improve social interaction and cognitive deficits in neonatal ventral hippocampal lesioned rats by indirectly restoring CaMKII signaling (43) at the opposite of the specific ways to potentially enhance CaMKII activity through gain of function-mutations (44).

Since a reduction in CaMKII activity may underly abnormal social behavior and the cognitive deficits associated with schizophrenia, we hypothesize that an the enhancement of CaMKII signaling could improve both social cognition and negative symptomatology in those living with this disorder (43) (Figure 2).

## Conclusion

Studies performed in patients with schizophrenia focused on the expression of CaMKII in *post-mortem* cerebral frontal cortex. Whereas α-and β-CaMKII protein expression were reported to be significantly reduced in this brain region (45), the expression of β-CaMKII mRNA has been shown to be significantly elevated (46). Additionally, it was reported that the prefrontal cortical expression of α-CaMKII mRNA is comparable in patients with schizophrenia and healthy control subjects (47). Recently, six mutations were found in the α-isoform of CaMKII in patients suffering from schizophrenia (48). Of these mutations, two CaMKII variants show impaired biochemical functions (48). Thus, CaMKII mutations causing impairments in CaMKII function can be a driver for schizophrenia in humans (48). In line with these findings and given that CaMKII is essential for learning and memory formation, several studies reported about new variants in the CaMKII genes that are linked to intellectual disability [for review: (44)]. Specifically, one *de novo* missense mutation in α-CaMKII was found in patients with autism (44), a disorder comprising social interaction and communication deficits (39). Interestingly, mice carrying the same mutation in α-CaMKII display reduced α-CaMKII protein forebrain levels and deficits in social interactions (39).

These assumptions are in agreement with studies performed in rodents. Indeed, the heterozygous CaMKII knockout, the neonatal ventral hippocampus lesion, and the NMDA-antagonism based models of schizophrenia show decreased CaMKII activity associated to a schizophrenia-like profile, thereby suggesting CaMKII as a potential therapeutic target in schizophrenia. Moreover, preclinical rodent studies enhancing CaMKII activity have demonstrated a potential for the treatment of social and cognitive impairments in schizophrenia (43), previously showing resistance to antipsychotics. This resistance to antipsychotics might be due to the fact that repeated treatment with antipsychotics decreases α-CaMKII protein levels in the striatum (49). Therefore, it is plausible that boosting CaMKII activity in a manner that counteracts this neuropsychiatric disease without disrupting the normal functioning of the brain, might restore this unmet need in the treatment of schizophrenia-like symptoms.

## Author Contributions

RE, IA, and AH wrote and edited the manuscript. All authors contributed to the article and approved the submitted version.

## Conflict of Interest

The authors declare that the research was conducted in the absence of any commercial or financial relationships that could be construed as a potential conflict of interest.

## Publisher’s Note

All claims expressed in this article are solely those of the authors and do not necessarily represent those of their affiliated organizations, or those of the publisher, the editors and the reviewers. Any product that may be evaluated in this article, or claim that may be made by its manufacturer, is not guaranteed or endorsed by the publisher.
